# Non-lethal effects of entomopathogenic nematode infection

**DOI:** 10.1038/s41598-021-96270-2

**Published:** 2021-08-24

**Authors:** Camila C. Filgueiras, Denis S. Willett

**Affiliations:** 1 Natural Enemy Management and Applications (NEMA) Lab, Department of Biology, UNC Asheville, Asheville, USA; 2 Applied Chemical Ecology Technology (ACET) Lab, Cornell AgriTech, Cornell University, Ithaca, USA

**Keywords:** Behavioural ecology, Community ecology, Antimicrobial responses

## Abstract

Entomopathogenic nematodes are typically considered lethal parasites of insect hosts. Indeed they are employed as such for biological control of insect pests. The effects of exposure to entomopathogenic nematodes are not strictly limited to mortality, however. Here we explore non-lethal effects of exposure to entomopathogenic nematodes by introducing the relatively non-susceptible pupal stage of *Delia antiqua* to thirteen different strains. We specifically chose to inoculate the pupal stage because it tends to be more resistant to infection, yet resides in the soil where it could come into contact with EPN biological control agents. We find that there is no significant mortality at the pupal stage, but that there are a host of strain-dependent non-lethal effects during and after the transition to adulthood including altered developmental times and changes in risk of death compared to controls. We also find that exposure to specific strains can reduce risk of mortality. These results emphasize the strain-dependent nature of entomopathogenic nematode infection and highlight the positive and negative ramifications for non-lethal effects for biological control of insect pests. Our work emphasizes the need for strain-specific screening of biological control agents before wide-spread adoption.

## Introduction

Entomopathogenic nematodes (EPNs) are highly effective parasites of insects^1^. Often attacking and infecting larval stages of soil–borne insects, EPN infective juveniles seek out, infect, and ultimately kill their insect hosts with the help of symbiotic bacteria^1,2^. Because these infective juveniles are often lethal and kill their insect hosts within 24–48 h, they make ideal biological control agents^2,3^.

For biological control, the lethal nature of entomopathogenic nematodes is desirable^3^. It is precisely this characteristic that make them successful in controlling agricultural pests. *Steinernema riobrave* Cabanillas, Raulston & Poinar and *Heterorhabditis bacteriophora* Poinar (Rhabditida, Heterorhabditidae) reduced populations of *Diaprepes* root weevil (*D. abbreviatus* L.) by up to 90%^4–6^. Strains of *S. scarabaei* (Stock and Koppenhöfer) and *H. zealandica* Poinar were effective in controlling grubs including *Popillia japonica* (Coleoptera: Scarabaeidae), respectively^7,8^. Other agricultural pests have also been successfully controlled by applications of EPNs^9,10^.

While there have been notable successes in controlling insect pests through applications of entomopathogenic nematodes, it seems likely that non-lethal or sub-lethal effects of EPN infection could potentially have long-lasting impacts on insect hosts. Indeed, this has begun to be documented. Infection by EPNs elicits very specific immune responses in host insects^11,12^. These can range from humoral responses, to encapsulation and melanization^12–14^. These responses have a cost, however. The host insect must invest substantial amounts of time and energy and fending off an attack by these parasites.

Even if the host insect is able to fend off attack, they may suffer fitness consequences. Colorado potato beetles infected with *Steinernema carpocapsae* (Weiser) as last instar larvae showed adult discoloration and deformation of the wings^15^. Colorado potato beetles exposed to *S. feltiae* demonstrated delayed metamorphosis^16^. These fitness consequences are not necessarily uniform in all environments, however. Abiotic factors such as temperature can affect both the susceptibility of insect immune system to parasite attack^17–19^ and the host-seeking behavior of parisitic nematodes^20^. Despite this variation, in each of these cases, EPNs had distinct consequences for the life history of their host insects; discoloration, deformation, and delayed metamorphosis presumably will negatively impact the ability of these insects to reproduce.

While some limited documentation of these non-lethal and sub-lethal effects of EPN infection is available, this is not the whole story. There is tremendous variation both in the ability of EPN strains to overcome host insect defenses and the ability of host insects to mount an effective immune response. Adult pine weevils that survived EPN exposure, for example, had very little if any impacts on feeding ability or other sub-lethal effects^21^. Indeed, encapsulated dead and live nematodes were recovered from infected insect hosts^21^. This is not an isolated report; specific strains of EPN can modulate and even escape host insect immune responses^11^. *Steinernema carpocapsae* can strongly inhibit the host prophenoloxidase–phenoloxidase system in red palm weevils and completely escape the encapsulation response^22^. In comparing multiple strains across four different insects, *S. glaseri* NC strain suppressed immune responses while *S. glaseri* FL strain was less successful^11^.

This strain dependent nature of host immune response has ramifications both for our understanding the nature of insect parasite infection, but also for the use of EPNs as biological control agents in applied systems—particularly in cases where insect life stages accessible to EPNs are more protected. Maggot flies of vegetables, for example, have pupal life stages that can reside in the soil for some time, but more susceptible larval stages tend to be more protected as they feed on the plant. With onion maggot (*Delia antiqua*) for instance, EPN susceptible larvae spend much of their life inside an onion bulb while pupae are more exposed in the soil. This pest can be a management challenge, particularly in areas of continuous cultivation; producers are actively seeking alternative management options^23,24^.

To evaluate the impact of entomopathogenic nematode exposure on the *D. antiqua* pupal life stage, we exposed pupae to 13 different EPN strains and closely monitored the results. Specifically we were interested in the strain dependent impact on long-term survival, mortality (if any), time spent in the pupal life stage, and long-lasting effects on adult fitness.

**Table 1:** The thirteen strains of entomopathogenic nematodes used in this trial. Note that *Steinernema khuongi* is a relatively newly identified *Steinernema* species^34^.

Species	Strain ID
*Heterorhabditis bacteriophora*	88
*Heterorhabditis bacteriophora*	NY
*Heterorhabditis indica*	LW
*Heterorhabditis indica*	BOB
*Steinernema carpocapsae*	
*Steinernema carpocapsae*	NY
*Steinernema diaprepesi*	BRT
*Steinernema diaprepesi*	HK31
*Steinernema feltiae*	NY
*Steinernema glaseri*	NC
*Steinernema riobrave*	
*Steinernema khuongi*	Web
*Steinernema khuongi*	ARCA

## Methods

### Organisms

#### Entomopathogenic nematodes

Thirteen strains of entomopathogenic nematodes were maintained in the Natural Enemy Management and Applications (NEMA) Lab at Cornell AgriTech (Table 1). These entomopathogenic nematodes were isolated from field locations across the eastern United States and reared in *Galleria mellonella* larvae following standard techniques^25^. To do so, 1 mL of EPN suspension (at 1000 IJ/mL) from each strain was added to five *G. mellonella* larvae placed on moistened filter paper in 54 mm petri dishes. Strict sterilization protocols were put in place to ensure strain isolation and prevent cross contamination. Dishes with inoculated *G. mellonella* were kept in a growth chamber at 25 $^{\circ }$C for 7 days, then they were transferred to White traps^26^. The filter paper under the worms were moisturized periodically to induce nematode release and migration to the water in the White traps. Following emergence from *G. mellonella* cadavers, nematodes were stored in 250ml tissue culture flasks at 500 IJs/ml at 25 $^{\circ }$C until use in assays within 7 days of emergence.

#### *Delia antiqua*

Larval *D. antiqua* were collected from infested onions in onion muck fields in upstate NY. Larvae were removed from the infested onions and placed in sand containers where they were allowed to pupate and emerge as adults. Adults were placed in plexiglass cages (40 cm $\times$ 40 cm) with a screening on two sides and a sleeve on the front and fed with a mix of sugar, skim dry milk, brewer’s yeast, and soy peptone (10:10:1:1); water was also provided through dental wicking immersed in a water flask. The cages were kept in an environmental chamber at 25 $^{\circ }$C, 16h of light and 8 h of dark, and 62% relative humidity. The food mixture and water flask was changed every 4 days. A plastic container with moistened sand and a halved organic yellow onion was added inside the cages for oviposition. Thereafter, the oviposition box was transferred to holding cages for eclosion, larval development, and pupal formation. Pupal formation occurs approximately 20 days after transfer of the oviposition box to the holding cage.

### Bioassays and experimental design

Early stage *D. antiqua* pupae were collected from 20 days after oviposition, then transferred to 24 well ELISA plates, one pupa per well. Each well also contained a filter paper disk. Each plate was divided in half to accommodate control and an EPN strain application in each plate: 12 wells each for control and 12 wells for EPN strain application. This allowed for a paired experimental design; comparisons of EPN treated pupa with controls were made within the same cohort in the same experimental environment. After adding one pupa in each well, 100 of solution was added to each pupa containing well. For control wells, this solution was DI water only. For EPN strain treated wells, this solution contained 100 infective juveniles of the respective strain.

After inoculation, ELISA plates containing inoculated pupae were kept in a chamber at $25 \pm 1\,^{\circ }{\text {C}}$, with a photoperiod of 16 h of light and 8 h of dark, and 62% RH. The plates were evaluated every 24 h beginning 6 days after inoculation when adults began to emerge until complete emergence and death of the adults. Adults were considered to have emerged when they were entirely out of the pupa shell, and dead when they did not respond to any stimulus when poked with a scalpel.

### Analysis

To evaluate the effect of differential EPN strain infection on *D. antiqua* pupae, we evaluated survival across the experimental time course and bootstrapped percent eclosion rates to determine direct EPN mortality at the pupal stage. Time (days) as pupae was modeled using Poisson regression with post-hoc mean differentiation using Dunnett’s test. Conformance to assumptions of normality and homoscedasticity were evaluated through examination of model diagnostics. Best fit models were chosen using a combination of model performance metrics including information criteria, pseudo-R^2^ values, and examination of residuals.

Logistic regression was used to evaluate the probability of adult mortality over time. Treatment, plate, and days since inoculation were factors considered in this analysis with response being the binary alive versus dead daily assessment. Post-hoc analysis was conducted with Dunnett’s test of treatment comparisons to control. Best fit models were chosen through a combination of model performance metrics including best-fit assessments, information criteria, pseudo-R^2^ values, and other model diagnostics including residual analysis.

All data were stored in flat CSV files and imported into R version 4.0.4 for analysis^27^. The *tidyverse*, *car*, and *emmeans* packages were used to facilitate analysis and graphical display of results^28–30^. All data and scripts for analysis will be available on GitHub following acceptance of this manuscript: https://github.com/acetworld/non-lethal-epn.

All methods complied with relevant jurisdictional guidelines and legislation.

### Signed/written informed consent

Landowners of field sites gave explicit permission to both work on their land, collect samples, and publish the results.

## Results

Inoculating pupae with a spectrum of entomopathogenic nematodes strains had differential effects on survival, time spent as a pupae, and likelihood of mortality as an adult.

**Figure 1:**
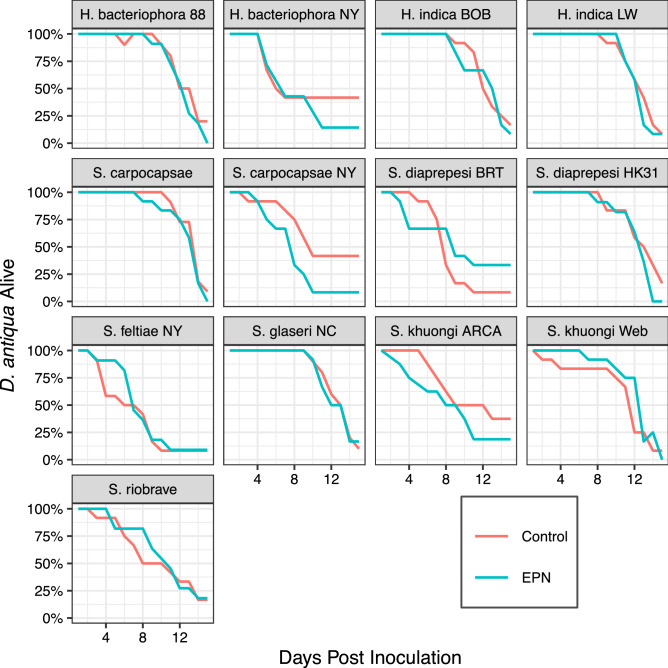
*D. antiqua* Survival following exposure to strains of entomopathogenic nematodes as pupae. Long-term survival appears to vary in a strain-dependent manner. Certain species seem to be more virulent and cause higher mortality in adults.

**Figure 2:**
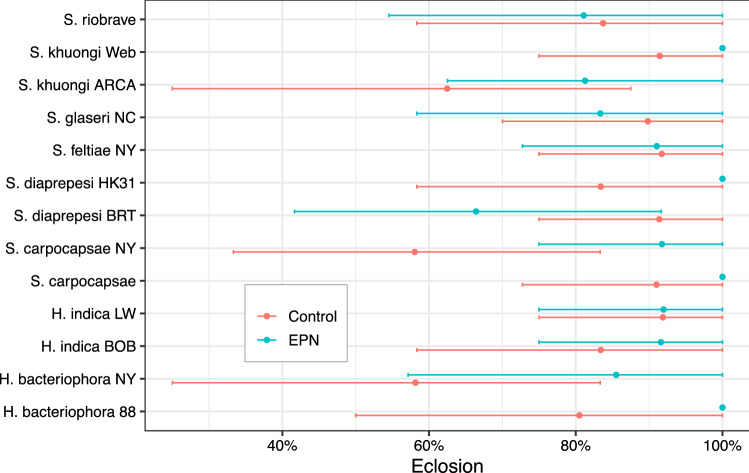
*D. antiqua* pupal eclosion following exposure to strains of entomopathogenic nematodes. The percentage of eclosion was high throughout the trial; most pupae successfully eclosed and made it to the adult lifestage. Inoculation with entomopathogenic nematodes did not cause significant mortality at the pupal nor prevent eclosion. There was no significant effect of strain. Percent eclosion denotes percent of cohort of pupae successfully eclosing to adulthood. Points and errorbars denote mean and bootstrapped 95% confidence intervals respectively.

**Figure 3:**
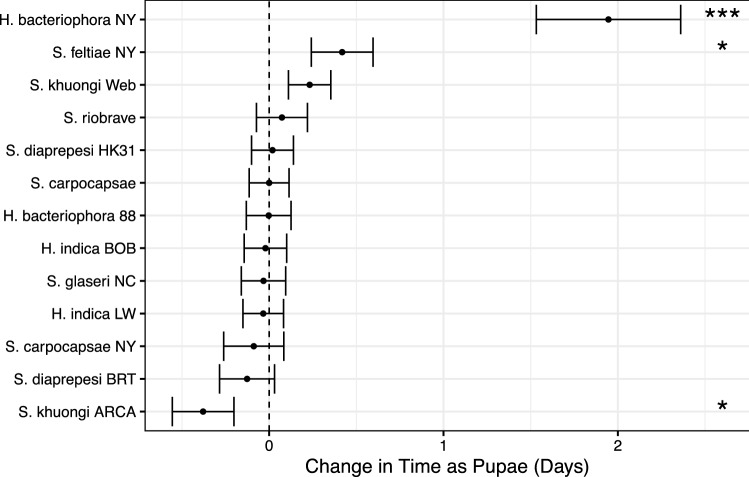
Change in time *D. antiqua* spent as pupae as compared to paired controls not inoculated with EPN strains. Points and error bars denote means and standard error respectively. Asterisks (*, **, and ***) denote significance at $p < 0.05$, $p < 0.01$, and $p < 0.001$ respectively.

**Table 2:** Results of modeling time as pupae and adult mortality risk. LRT denotes likelihood ratio test. Pseudo $R^2$ calculated using Nagelkerke’s method^35^.

Time as Pupae				Adult Mortality Risk			
Factor	$\chi ^2$	*df*	*P*	Factor	$\chi ^2$	*df*	*P*
Treatment	3.691	1	0.055	Treatment	8.5	1	0.003
Strain	188.6	12	< 0.001	Strain	292.28	12	< 0.001
Treatment $\times$ Strain	45.1	12	<0.001	Time	2031.9	1	< 0.001
				Treatment $\times$ Strain	71.06	12	< 0.001
LRT	289.42	25	< 0.001	LRT	2370.4	26	< 0.001
Pseudo-$R^2$	0.68			Pseudo-$R^2$	57.5		

Pupal infection by some strains of EPN had little to no effect on long-term survival of *D. antiqua* (Fig. 1A). Survival curves for *D. antiqua* inoculated with *H. indica* LW, *S. carpocapsae*, and *S. glaseri* NC were similar with non-inoculated controls. On the other hand, infection by *S. diaprepesi* BRT seemed to prolong life; *D. antiqua* pupae infected with this strain maintained a survival rate greater than 25% and higher than controls through the end of the trial. Other strains, particularly the *S. carpocapsae* strain isolated from New York, were particularly virulent even against pupae (Fig. 1B).

Inoculating *D. antiqua* pupae with EPNs did not cause attributable immediate mortality at the pupal stage (Fig. 2). The overall eclosion rate from pupae to adults was 86% for the whole trial and many cohorts of both EPN treated and controls had 100% emergence. There were no significant ($p > 0.05$) effects of EPN inoculation on probability of mortality at the pupal life stage and no significant differences ($p > 0.05$) between EPN treated pupae and paired controls for all strains evaluated in this trial.

Inoculating *D. antiqua* pupae with EPNs altered the time spent as pupae in a strain-dependent manner (Fig. 3). Treatment with EPNs, EPN strain, and their interaction significantly explained observed differences in pupal time (Table 2). Treatments of *H. bacteriophora* and *S. feltiae* from NY significantly increased time (by $1.9\pm 0.4$ and $0.4\pm 0.2$ days on average respectively, $p < 0.01$) spent as pupae when compared with paired controls. Treatments with *S. khuongi* ARCA significantly reduced the time *D. antiqua* (by $0.3\pm 0.2$ days on average, $p = 0.03$) spent as pupae when compared with paired controls.

**Figure 4:**
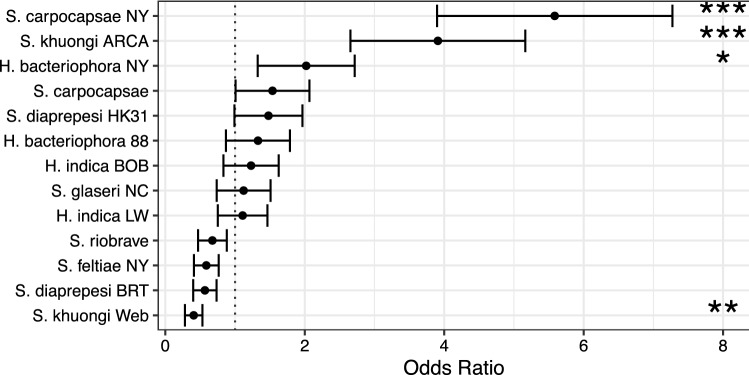
Odds of adult mortality following exposure to EPN strains as a pupa based on comparison to paired controls. Odds ratios significantly greater than 1 indicate and fold increase in probability of mortality. For example, adult *D. antiqua* exposed to *S. carpocapsae* NY as a pupae were  5 times more likely to die than paired controls under the same conditions who were not exposed. Odds ratios not significantly different than one indicate no change in odds of dying. Odds ratios significantly less than 1 (as is the case with *S. khuongi* Web indicate that exposure to entomopathogenic nematodes decreased the odds of dying. Points and error bars denote mean odds ratios and standard error respectively. Asterisks (*, **, and ***) denote a significant difference from 1 at $p < 0.05$, $p < 0.01$, and $p < 0.001$ respectively.

Exposure to EPNs during the pupal life stage significantly influenced the odds of mortality during the adult stage in an EPN strain-dependent manner (Fig. 4, Table 2). Inoculations with *S. carpocapsae* NY, *S. khuongi* ARCA, and *H. bacteriophora* NY significantly ($p < 0.04$) increased the odds of mortality of EPN exposure individuals as compared with controls. Individuals exposed to *S. carpocapsae* NY were $5.6\pm 1.7$ times more likely to die than those individuals in the paired control cohort. Exposure to *S. khuongi* Web significantly decreased the odds of mortality ($p = 0.003$); individuals exposed to this strain were $59.3\pm 12.6$% less likely to die as compared with individuals from the paired control cohort.

## Discussion

*D. antiqua* pupae exposed to entomopathogenic nematodes (EPNs) demonstrated effects that persisted across their adult life in a strain dependent manner. Echoing the results of Li et al.^11^, different strains, even of the same species, produced markedly different effects. These effects were predominantly non-lethal. While adult survival was variable (Fig. 1), exposure to EPNs did not kill the pupae nor result in substantial immediate mortality of adults. The majority of individual insects assayed in this trials eclosed as alive adults and there were no significant effects of EPN exposure on that eclosion (Fig. 2).

Eclosion and arrival at adulthood was not synchronous, however (Fig. 3). Exposure to EPNs as pupae altered the time individuals spent as pupae in a strain-dependent manner. Similar to results observed by Ebrahimi et al.^16^ in Colorado potato beetle, certain strains significantly increased the time spent as pupae. Unlike findings from previous work, exposure to *S. khuongi* ARCA sped up development. While these developmental changes were at most on the order of two days, this highlights the effect of exposure on lifecycle development and has possible ramifications for performance as adults.

The adults that eclosed were not uniformly healthy. While we did not make extensive and specific assessments of morphology, adult flies emerging from control groups seemed uniformly healthy. Deformities similar to those observed by^16^ and^15^ were noted in some adults in EPN exposed cohorts. These deformities likely contributed to early mortality and/or a higher likelihood of mortality observed in some EPN exposed cohorts. Exposure to *S. carpocapsae* NY and *S. khuongi* ARCA increased the odds that a given individual would die by approximately 5 and 4 fold respectively (Fig. 4).

Unexpectedly, we observed differential effects of exposure on different metrics by strain; the strains altering time spent as pupae were not consistently the same strains increasing the odds of mortality as an adult. For example, *S. carpocapsae* NY did not significantly alter time spent as a pupae, but substantially increased the odds of mortality. *H. bacteriophora* NY substantially increased the time spent as a pupae and slightly increased the odds of mortality as an adult, but *S. khuongi* ARCA reduced the time spent as a pupae and substantially increased odds of mortality as an adult. These contrasting results suggest competing underlying mechanisms of combating EPN infection on the part of the insect host or escaping immune responses on the part of EPNs.

An additional unexpected result was the ability of exposure to EPNs to reduce the odds of mortality as an adult. Exposure to *S. khuongi* Web reduced the odds of mortality of adults by almost 60%. While the mechanism behind this is unknown, this could be an example of eustress where stimulation of an immune response by *S. khuongi* Web actually improves the longevity and viability of adults. While more work would be needed to bear this out, it could align well with other research showing that stimulation of certain immune responses in humans and *C. elegans* can influence longevity^31,32^.

Irrespective of the mechanism, the example of *S. khuongi* Web and strain-dependent responses observed in this trial demonstrate the importance of strain considerations in working with nematode parasites of insects. Most of the literature documents positive effects of EPNs in increasing insect mortality alongside documentation of ineffective species and strains that do not substantially increase mortality. Our results suggest that in certain cases, with exposure to certain strains at specific life stages, EPNs may actually elicit immune responses that reduce the odds of mortality.

These immune responses seem to have long-lasting effects over an insect’s life and, for those strains that increase odds of mortality, hold great potential for biological control. Echoing other recent work, specific strains seem better able to overcome host immune responses, even in the pupal stage^33^. Despite a lack of immediate mortality in this trial during the pupal life stage and despite eclosion as alive adults, exposure to specific EPN strains during the pupal stage can increase the odds of death as an adult. This approach could make adults more susceptible to other mortality factors and, over the long term, lead to reduced populations. It is important to note, however, the importance of strain considerations in designing effective solutions for biological control. As our results suggest, while certain strains can certainly be more virulent, others may have very little effect or even have marginally beneficial effects on host insect populations.
